# Tetra­methyl­ammonium dimethyl (phenyl­sulfonyl­amido)phosphate(1−)

**DOI:** 10.1107/S1600536811055024

**Published:** 2012-01-07

**Authors:** Elizaveta A. Trush, Oleg V. Shishkin, Victor A. Trush, Irina S. Konovalova, Tetyana Yu. Sliva

**Affiliations:** a National Taras Shevchenko University, Department of Chemistry, Volodymyrska str. 64, 01601 Kyiv, Ukraine; b STC "Institute for Syngle Crystals", 60 Lenina ave., Khar’kov 61001, Ukraine

## Abstract

The title compound, C_4_H_12_N^+^·C_8_H_11_NO_5_PS^−^, was obtained from tetra­methyl­ammonium hydroxide and dimeth­yl(phenyl­sulfon­yl)amido­phosphate. The tetra­methyl­ammonium cation has a slightly distorted tetra­hedral configuration and the N—C bond lengths lie in the range 1.457 (3)–1.492 (3) Å. In the crystal, no classical hydrogen bonds are observed between the cation and the anion.

## Related literature

For the synthesis of sulfonyl­amide derivatives, see: Kirsanov & Shevchenko (1956 ▶); Pietraszkiewicz *et al.* (2002 ▶); Trush *et al.* (2009 ▶); Moroz *et al.* (2009 ▶); Shatrava *et al.* (2010 ▶). For the crystal structures of tetra­methyl­ammonium compounds, see: Cao *et al.* (2008 ▶); Liu *et al.* (2004 ▶).
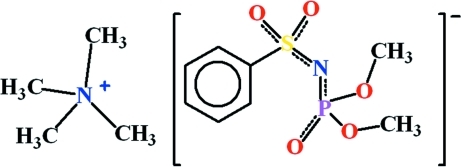


## Experimental

### 

#### Crystal data

C_4_H_12_N^+^·C_8_H_11_NO_5_PS^−^
*M* *_r_* = 338.36
Monoclinic, 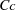

*a* = 15.2840 (9) Å
*b* = 9.269 (2) Å
*c* = 12.1650 (11) Å
β = 98.279 (9)°
*V* = 1705.4 (4) Å^3^
*Z* = 4
Mo *K*α radiation
μ = 0.30 mm^−1^
*T* = 293 K
0.40 × 0.20 × 0.10 mm

#### Data collection

Oxford Diffraction Xcalibur Sapphire3 diffractometer
Absorption correction: multi-scan (*CrysAlis PRO*; Oxford Diffraction, 2010 ▶) *T*_min_ = 0.582, *T*_max_ = 1.000
8644 measured reflections
3928 independent reflections
2406 reflections with *I* > 2σ(*I*)
*R*_int_ = 0.035

#### Refinement

*R*[*F* ^2^ > 2σ(*F* ^2^)] = 0.032
*wR*(*F* ^2^) = 0.063
*S* = 0.77
3928 reflections
197 parameters
2 restraints
H-atom parameters constrained
Δρ_max_ = 0.23 e Å^−3^
Δρ_min_ = −0.15 e Å^−3^
Absolute structure: Flack (1983 ▶), 1444 Friedel pairs
Flack parameter: 0.05 (6)

### 

Data collection: *CrysAlis CCD* (Oxford Diffraction, 2006 ▶); cell refinement: *CrysAlis CCD*; data reduction: *CrysAlis RED* (Oxford Diffraction, 2006 ▶); program(s) used to solve structure: *SHELXS97* (Sheldrick, 2008 ▶); program(s) used to refine structure: *SHELXL97* (Sheldrick, 2008 ▶); molecular graphics: *XP* within *SHELXTL* (Sheldrick, 2008 ▶); software used to prepare material for publication: *PLATON* (Spek, 2009 ▶).

## Supplementary Material

Crystal structure: contains datablock(s) I, global. DOI: 10.1107/S1600536811055024/wn2457sup1.cif

Structure factors: contains datablock(s) I. DOI: 10.1107/S1600536811055024/wn2457Isup2.hkl

Additional supplementary materials:  crystallographic information; 3D view; checkCIF report

## Figures and Tables

**Fig. 1.**
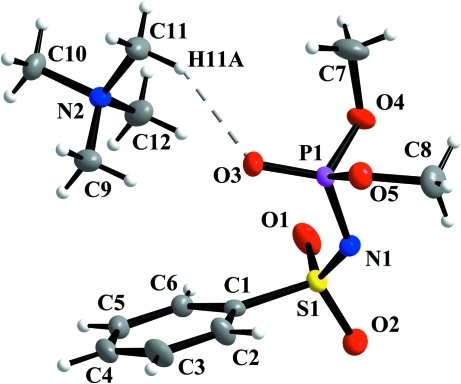
The asymmetric unit of dimethyl(phenylsulfonyl)amidophosphato- tetramethylammonium. Displacement ellipsoids are drawn at the 20% probability level; hydrogen atoms are drawn as spheres of arbitrary radius.

**Fig. 2.**
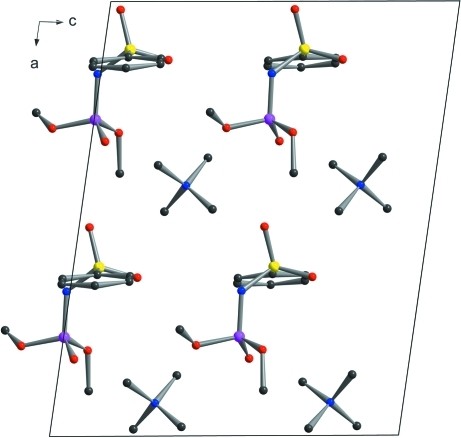
Projection of the structure {N(CH3)4+[C6H5SO2NP(O)(OCH3)2]-} on the ac plane.

## supplementary crystallographic information

### Comment

To date the coordination chemistry of phosphorylated sulphonylamide ligands has been of great interest (Pietraszkiewicz *et al.*, 2002). Earlier sulfonylamide derivatives of the general type RSO_2_NHP(O)(*R'*)_2_ and their coordination compounds have been systematically investigated and some results of our study have been already published (Moroz *et al.*, 2009, Shatrava *et al.*, 2010, Trush *et al.* 2009). However, there are no reports of the crystal structure of any alkali- or onic- salts of dimethyl(phenylsulphonyl)amidophosphate (HL). This paper reports the crystal structure of the compound {N(CH_3_)_4_^+^[C_6_H_5_SO_2_NP(O)(OCH_3_)_2_]^-^} (Fig. 1).

The highly polar anion contains six potential donor centers and, in spite of this fact, coordinated molecules of water or alcohol have not been detected. (Fig. 2).

The X-ray crystal structure reveals that there are no contacts shorter than 2.44 Å between cation and anion, (the presence of weak C—H···O contacts with the participation of PO and SO_2_ oxygens and protons of cation is observed), so the bonding may be considered as mainly ionic.

The dimethyl(phenylsulfonyl)amidophosphatotetramethylammonium salt includes a deprotonated sulphonylamidophosphate anion and cation; the latter has a standard N(CH_3_)_4_^+^-tetrahedral configuration (Fig. 1). The presence of non-equivalent values of C—N bond lengths is a general feature for earlier, structurally investigated tetramethylammonium compounds (Cao *et al.*, 2008; Liu *et al.*, 2004). The geometry of the nearest environment of the phosphorus atom in {L^-^} can be described as a distorted tetrahedron. The bond lengths P1—O3 and P1—N1 have values 1.460 (2) and 1.591 (2) Å, respectively, which are typical of phosphorylated sulfonylamide for compounds with ether-type substituents (Moroz *et al.*, 2009).

The fragments O2—S1—N1—P1 and S1—N1—P1—O5 are practically planar; the values the of corresponding torsion angles are -179.8 (1)° and 163.6 (1)°, respectively; the interplanar angle is 13.2 (2)°. The average deviations of these atoms from these planes do not exceed 0.001 (4) and 0.08 (6) Å, respectively. The phenyl ring is rotated to a considerable extent with respect to the S—N bond, the value of the C2—C1—S1—N1 torsion angle being -36.1 (3)°.

### Experimental

The dimethyl(phenylsulfonyl)amidophosphate (HL) was prepared according to the earlier published procedures (Kirsanov *et al.*, 1956). The tetramethylammonium salt {N(CH_3_)_4_^+^[C_6_H_5_SO_2_NP(O)(OCH_3_)_2_]^-^} was obtained by the neutralization reaction: tetramethylammonium hydroxide (0.365 g, 1 mmol of 25% solution) was added dropwise to an equimolar amount (0.265 g, 1 mmol) of HL which was dissolved in 10 ml of isopropanol. The completion of the reaction was checked with phenolphthalein. Colorless crystals suitable for X-ray diffraction were separated. The yield was 0.31 g (92% starting from HL).

### Refinement

All H atoms were positioned geometrically and refined using a riding model, with C—H = 0.93–0.96 Å and *U*_iso_(H) = x*U*_eq_(C), where x = 1.5 for methyl H and 1.2 for all other H atoms. A rotating-group model was applied for the methyl groups. The absolute configuration was determined and the Flack parameter refined to 0.05 (6).

### Crystal data

**Table:**

C_4_H_12_N^+^·C_8_H_11_NO_5_PS^−^	*F*(000) = 720
*M**_r_* = 338.36	*D*_x_ = 1.318 Mg m^−^^3^
Monoclinic, *C**c*	Mo *K*α radiation, λ = 0.71073 Å
*a* = 15.2840 (9) Å	Cell parameters from 2883 reflections
*b* = 9.269 (2) Å	θ = 3.0–32.2°
*c* = 12.1650 (11) Å	µ = 0.30 mm^−^^1^
β = 98.279 (9)°	*T* = 293 K
*V* = 1705.4 (4) Å^3^	Needle, colourless
*Z* = 4	0.40 × 0.20 × 0.10 mm

### Data collection

**Table:**

Oxford Diffraction Xcalibur Sapphire3 diffractometer	3928 independent reflections
Radiation source: fine-focus sealed tube	2406 reflections with *I* > 2σ(*I*)
graphite	*R*_int_ = 0.035
Detector resolution: 16.1827 pixels mm^-1^	θ_max_ = 30.0°, θ_min_ = 3.0°
ω scans	*h* = −21→20
Absorption correction: multi-scan (CrysAlis PRO; Oxford Diffraction, 2010)	*k* = −12→13
*T*_min_ = 0.582, *T*_max_ = 1.000	*l* = −17→17
8644 measured reflections	

### Refinement

**Table:**

Refinement on *F*^2^	Hydrogen site location: inferred from neighbouring sites
Least-squares matrix: full	H-atom parameters constrained
*R*[*F*^2^ > 2σ(*F*^2^)] = 0.032	*w* = 1/[σ^2^(*F*_o_^2^) + (0.0302*P*)^2^] where *P* = (*F*_o_^2^ + 2*F*_c_^2^)/3
*wR*(*F*^2^) = 0.063	(Δ/σ)_max_ < 0.001
*S* = 0.77	Δρ_max_ = 0.23 e Å^−^^3^
3928 reflections	Δρ_min_ = −0.15 e Å^−^^3^
197 parameters	Extinction correction: *SHELXL97* (Sheldrick, 2008), Fc^*^=kFc[1+0.001xFc^2^λ^3^/sin(2θ)]^-1/4^
2 restraints	Extinction coefficient: 0.0053 (4)
Primary atom site location: structure-invariant direct methods	Absolute structure: Flack (1983), 1444 Friedel pairs
Secondary atom site location: difference Fourier map	Flack parameter: 0.05 (6)

### Special details

**Table:**

Experimental. Analysis found: IR (KBr pellet, cm^-1^): 1245(ν), 1220(νs), 1190(νs), 1060(νs), 1040(δ); 1060 (s, SO_2_) and 1190 (s, PO). ^1^H NMR (DMSO-d^6^): 3.4 d 6H, ^3^*J*_PH_ =11.6 Hz; 7.78 dd (α), 2H; 7.38 m (β+γ), 3H; ^31^P (DMSO-d^6^) -3.27 h, ^3^*J*_HP_ = 11.6 Hz.
Geometry. All esds (except the esd in the dihedral angle between two l.s. planes) are estimated using the full covariance matrix. The cell esds are taken into account individually in the estimation of esds in distances, angles and torsion angles; correlations between esds in cell parameters are only used when they are defined by crystal symmetry. An approximate (isotropic) treatment of cell esds is used for estimating esds involving l.s. planes.
Refinement. Refinement of F^2^ against ALL reflections. The weighted R-factor wR and goodness of fit S are based on F^2^, conventional R-factors R are based on F, with F set to zero for negative F^2^. The threshold expression of F^2^ > 2sigma(F^2^) is used only for calculating R-factors(gt) etc. and is not relevant to the choice of reflections for refinement. R-factors based on F^2^ are statistically about twice as large as those based on F, and R- factors based on ALL data will be even larger.

### Fractional atomic coordinates and isotropic or equivalent isotropic displacement parameters (Å^2^)

**Table:**

	*x*	*y*	*z*	*U*_iso_*/*U*_eq_	
P1	−0.72846 (3)	−0.17887 (6)	−0.99705 (4)	0.03893 (14)	
S1	−0.88898 (4)	−0.24929 (6)	−0.92536 (5)	0.04416 (14)	
N1	−0.83352 (11)	−0.18063 (19)	−1.00894 (14)	0.0476 (4)	
N2	−0.57619 (11)	−0.5029 (2)	−0.71028 (15)	0.0466 (5)	
O1	−0.86516 (14)	−0.20683 (19)	−0.81208 (13)	0.0773 (6)	
O2	−0.98092 (11)	−0.22632 (19)	−0.96904 (16)	0.0740 (5)	
O3	−0.67793 (12)	−0.30800 (18)	−0.96015 (16)	0.0681 (5)	
O4	−0.69770 (10)	−0.04211 (17)	−0.92503 (14)	0.0618 (4)	
O5	−0.70883 (10)	−0.14025 (18)	−1.11691 (12)	0.0586 (4)	
C1	−0.87219 (12)	−0.4386 (2)	−0.92758 (17)	0.0373 (5)	
C2	−0.86269 (19)	−0.5079 (3)	−1.0247 (2)	0.0609 (6)	
H2	−0.8643	−0.4558	−1.0902	0.073*	
C3	−0.8507 (2)	−0.6556 (3)	−1.0247 (2)	0.0725 (8)	
H3	−0.8449	−0.7025	−1.0908	0.087*	
C4	−0.84735 (16)	−0.7336 (3)	−0.9293 (2)	0.0635 (7)	
H4	−0.8390	−0.8329	−0.9298	0.076*	
C5	−0.85644 (15)	−0.6633 (3)	−0.8328 (2)	0.0567 (6)	
H5	−0.8545	−0.7152	−0.7671	0.068*	
C6	−0.86836 (14)	−0.5174 (3)	−0.83254 (17)	0.0474 (6)	
H6	−0.8740	−0.4709	−0.7662	0.057*	
C7	−0.60472 (19)	−0.0089 (4)	−0.9058 (3)	0.1007 (12)	
H7A	−0.5927	0.0693	−0.9531	0.151*	
H7B	−0.5715	−0.0923	−0.9218	0.151*	
H7C	−0.5880	0.0184	−0.8295	0.151*	
C8	−0.7451 (2)	−0.0104 (3)	−1.1696 (2)	0.0815 (9)	
H8B	−0.7178	0.0719	−1.1307	0.122*	
H8A	−0.8077	−0.0078	−1.1680	0.122*	
H8C	−0.7339	−0.0084	−1.2453	0.122*	
C9	−0.62334 (17)	−0.5989 (3)	−0.7979 (2)	0.0644 (7)	
H9B	−0.6614	−0.6634	−0.7650	0.097*	
H9A	−0.5809	−0.6538	−0.8314	0.097*	
H9C	−0.6581	−0.5414	−0.8535	0.097*	
C10	−0.52163 (19)	−0.5918 (3)	−0.6247 (2)	0.0697 (7)	
H10B	−0.4877	−0.5297	−0.5716	0.105*	
H10A	−0.4824	−0.6516	−0.6597	0.105*	
H10C	−0.5595	−0.6515	−0.5876	0.105*	
C11	−0.51646 (17)	−0.4051 (3)	−0.7616 (2)	0.0689 (7)	
H11C	−0.4744	−0.4614	−0.7947	0.103*	
H11B	−0.4857	−0.3433	−0.7056	0.103*	
H11A	−0.5507	−0.3475	−0.8177	0.103*	
C12	−0.63993 (19)	−0.4189 (3)	−0.6587 (2)	0.0727 (8)	
H12C	−0.6751	−0.3617	−0.7142	0.109*	
H12B	−0.6091	−0.3567	−0.6030	0.109*	
H12A	−0.6776	−0.4830	−0.6249	0.109*	

### Atomic displacement parameters (Å^2^)

**Table:**

	*U*^11^	*U*^22^	*U*^33^	*U*^12^	*U*^13^	*U*^23^
P1	0.0396 (3)	0.0345 (3)	0.0437 (3)	−0.0002 (3)	0.0096 (2)	0.0012 (3)
S1	0.0473 (3)	0.0415 (3)	0.0467 (3)	−0.0013 (2)	0.0171 (2)	0.0014 (3)
N1	0.0389 (10)	0.0518 (11)	0.0528 (11)	−0.0007 (8)	0.0085 (8)	0.0104 (9)
N2	0.0414 (11)	0.0503 (12)	0.0489 (11)	−0.0025 (9)	0.0087 (8)	0.0038 (9)
O1	0.1328 (17)	0.0603 (11)	0.0427 (10)	−0.0138 (11)	0.0264 (10)	−0.0104 (8)
O2	0.0463 (10)	0.0711 (11)	0.1084 (15)	0.0072 (8)	0.0238 (9)	0.0168 (10)
O3	0.0553 (11)	0.0492 (10)	0.0971 (14)	0.0065 (8)	0.0017 (9)	0.0155 (9)
O4	0.0595 (11)	0.0609 (11)	0.0665 (11)	−0.0170 (8)	0.0142 (8)	−0.0184 (9)
O5	0.0662 (11)	0.0632 (11)	0.0513 (10)	0.0048 (8)	0.0248 (8)	0.0029 (8)
C1	0.0332 (11)	0.0441 (11)	0.0348 (11)	−0.0091 (8)	0.0064 (8)	−0.0025 (10)
C2	0.0954 (19)	0.0482 (14)	0.0417 (13)	−0.0123 (13)	0.0188 (12)	−0.0044 (11)
C3	0.101 (2)	0.0536 (17)	0.0659 (18)	−0.0144 (14)	0.0224 (15)	−0.0256 (14)
C4	0.0629 (16)	0.0392 (14)	0.089 (2)	−0.0055 (11)	0.0140 (14)	−0.0010 (15)
C5	0.0580 (16)	0.0503 (15)	0.0607 (16)	−0.0055 (11)	0.0047 (12)	0.0146 (12)
C6	0.0552 (14)	0.0525 (14)	0.0344 (13)	−0.0052 (11)	0.0059 (10)	0.0045 (10)
C7	0.073 (2)	0.102 (3)	0.124 (3)	−0.0374 (19)	0.0050 (19)	−0.027 (2)
C8	0.101 (2)	0.081 (2)	0.0664 (18)	0.0027 (17)	0.0261 (16)	0.0275 (15)
C9	0.0523 (15)	0.0679 (16)	0.0732 (17)	−0.0069 (12)	0.0099 (13)	−0.0097 (14)
C10	0.0601 (15)	0.0793 (18)	0.0679 (18)	0.0110 (14)	0.0030 (12)	0.0266 (14)
C11	0.0565 (16)	0.0727 (18)	0.0772 (19)	−0.0160 (13)	0.0083 (13)	0.0213 (14)
C12	0.0573 (15)	0.0800 (19)	0.081 (2)	0.0114 (15)	0.0116 (13)	−0.0142 (16)

### Geometric parameters (Å, °)

**Table:**

P1—O3	1.4597 (17)	C5—C6	1.365 (3)
P1—O5	1.5718 (16)	C5—H5	0.9300
P1—O4	1.5738 (16)	C6—H6	0.9300
P1—N1	1.5915 (17)	C7—H7A	0.9600
S1—O1	1.4290 (16)	C7—H7B	0.9600
S1—O2	1.4447 (16)	C7—H7C	0.9600
S1—N1	1.5510 (18)	C8—H8B	0.9600
S1—C1	1.774 (2)	C8—H8A	0.9600
N2—C12	1.457 (3)	C8—H8C	0.9600
N2—C10	1.486 (3)	C9—H9B	0.9600
N2—C11	1.486 (3)	C9—H9A	0.9600
N2—C9	1.492 (3)	C9—H9C	0.9600
O4—C7	1.440 (3)	C10—H10B	0.9600
O5—C8	1.437 (3)	C10—H10A	0.9600
C1—C6	1.362 (3)	C10—H10C	0.9600
C1—C2	1.370 (3)	C11—H11C	0.9600
C2—C3	1.381 (4)	C11—H11B	0.9600
C2—H2	0.9300	C11—H11A	0.9600
C3—C4	1.361 (4)	C12—H12C	0.9600
C3—H3	0.9300	C12—H12B	0.9600
C4—C5	1.367 (3)	C12—H12A	0.9600
C4—H4	0.9300		
			
O3—P1—O5	107.99 (10)	C5—C6—H6	119.4
O3—P1—O4	112.76 (10)	O4—C7—H7A	109.5
O5—P1—O4	104.56 (9)	O4—C7—H7B	109.5
O3—P1—N1	120.12 (10)	H7A—C7—H7B	109.5
O5—P1—N1	104.05 (9)	O4—C7—H7C	109.5
O4—P1—N1	105.99 (9)	H7A—C7—H7C	109.5
O1—S1—O2	114.42 (12)	H7B—C7—H7C	109.5
O1—S1—N1	115.58 (11)	O5—C8—H8B	109.5
O2—S1—N1	107.03 (10)	O5—C8—H8A	109.5
O1—S1—C1	105.65 (11)	H8B—C8—H8A	109.5
O2—S1—C1	105.98 (10)	O5—C8—H8C	109.5
N1—S1—C1	107.57 (10)	H8B—C8—H8C	109.5
S1—N1—P1	125.74 (11)	H8A—C8—H8C	109.5
C12—N2—C10	109.7 (2)	N2—C9—H9B	109.5
C12—N2—C11	110.1 (2)	N2—C9—H9A	109.5
C10—N2—C11	108.40 (18)	H9B—C9—H9A	109.5
C12—N2—C9	109.99 (19)	N2—C9—H9C	109.5
C10—N2—C9	109.5 (2)	H9B—C9—H9C	109.5
C11—N2—C9	109.07 (19)	H9A—C9—H9C	109.5
C7—O4—P1	118.14 (16)	N2—C10—H10B	109.5
C8—O5—P1	119.47 (16)	N2—C10—H10A	109.5
C6—C1—C2	118.9 (2)	H10B—C10—H10A	109.5
C6—C1—S1	120.41 (16)	N2—C10—H10C	109.5
C2—C1—S1	120.68 (17)	H10B—C10—H10C	109.5
C1—C2—C3	119.7 (2)	H10A—C10—H10C	109.5
C1—C2—H2	120.2	N2—C11—H11C	109.5
C3—C2—H2	120.2	N2—C11—H11B	109.5
C4—C3—C2	121.0 (2)	H11C—C11—H11B	109.5
C4—C3—H3	119.5	N2—C11—H11A	109.5
C2—C3—H3	119.5	H11C—C11—H11A	109.5
C3—C4—C5	118.9 (2)	H11B—C11—H11A	109.5
C3—C4—H4	120.6	N2—C12—H12C	109.5
C5—C4—H4	120.6	N2—C12—H12B	109.5
C6—C5—C4	120.3 (2)	H12C—C12—H12B	109.5
C6—C5—H5	119.9	N2—C12—H12A	109.5
C4—C5—H5	119.9	H12C—C12—H12A	109.5
C1—C6—C5	121.2 (2)	H12B—C12—H12A	109.5
C1—C6—H6	119.4		
